# An Activin Receptor IA/Activin-Like Kinase-2 (R206H) Mutation in Fibrodysplasia Ossificans Progressiva

**DOI:** 10.1155/2013/260371

**Published:** 2013-04-09

**Authors:** Rafael Herrera-Esparza, Deyanira Pacheco-Tovar, Juan José Bollain-y-Goytia, Felipe Torres del Muro, Roxana Ramírez-Sandoval, María Guadalupe Pacheco-Tovar, María Castañeda-Ureña, Esperanza Avalos-Díaz

**Affiliations:** ^1^Departments of Immunology and Molecular Biology, School of Biology, Universidad Autónoma de Zacatecas, Chepinque 306, Colonia Lomas de la Soledad, 98040 Zacatecas, ZAC, Mexico; ^2^Department of Rheumatology, Hospital Civil de Guadalajara, 44280 Guadalajara, JAL, Mexico

## Abstract

Fibrodysplasia ossificans progressiva (FOP) is an exceptionally rare genetic disease that is characterised by congenital malformations of the great toes and progressive heterotopic ossification (HO) in specific anatomical areas. This disease is caused by a mutation in activin receptor IA/activin-like kinase-2 (ACVR1/ALK2). A Mexican family with one member affected by FOP was studied. The patient is a 19-year-old female who first presented with symptoms of FOP at 8 years old; she developed spontaneous and painful swelling of the right scapular area accompanied by functional limitation of movement. Mutation analysis was performed in which genomic DNA as PCR amplified using primers flanking exons 4 and 6, and PCR products were digested with *Cac8I* and *HphI* restriction enzymes. The most informative results were obtained with the exon 4 flanking primers and the *Cac8I* restriction enzyme, which generated a 253 bp product that carries the ACVR1 617G>A mutation, which causes an amino acid substitution of histidine for arginine at position 206 of the glycine-serine (GS) domain, and its mutation results in the dysregulation of bone morphogenetic protein (BMP) signalling that causes FOP.

## 1. Introduction 

Bone morphogenetic protein (BMP) is a highly conserved molecule that regulates the BMP receptor signalling pathway and plays an important role in morphogenesis during vertebrate development and in adults [1]. There are two serine/threonine kinase type BMP receptors, both of which have similar functional glycine-serine (GS) domains that are critical for signal transduction. ACVR1/ALK2 is a type I receptor that is expressed in many tissues, including skeletal muscles and chondrocytes. The constitutive activation of ACVR1/ALK2 induces alkaline phosphatase activity in C2C12 inmortal line of mouse skeletal myoblasts cells, resulting in the upregulation of BMP4 and the downregulation of BMP antagonists. ACVR1/ALK2 is an activin-like kinase and is one of the six activin kinase genes that encode transforming growth factor-beta (TGF-*β*)/BMP type I serine/threonine transmembrane receptors, all of which are involved in cell fate and differentiation. In patients with fibrodysplasia ossificans progressiva (FOP), the mutated ACVR1/ALK2 receptor is hyperactive, which results in the expansion of cartilaginous elements and ectopic chondrogenesis and promotes joint ankylosis. FOP is caused by a heterozygous missense activating mutation (c.617G>A; R206H) that affects the glycine-serine (GS) activation domain of ACVR1, which is essential for signal transduction [2, 3], but it is not the only mutation. We present the clinical and genetic analysis of a patient with FOP. 

## 2. Case Presentation

**Figure 1:**
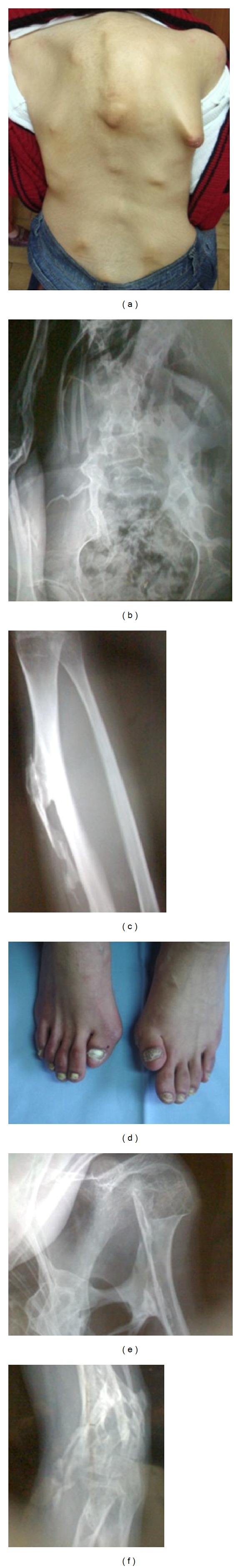
Clinical photograph. (a) Heterotopic ossification of the right scapular area, which functionally limited patient movement. Note the presence of pseudotumours of soft tissue at different locations in the back. (b) Column radiography showing abnormal calcification and “pseudoexostosis” indicating ligament ossification at the site of attachment. (c) Right forearm with cubit exostoses dependent on ligaments and aponeuroses. (d) Congenital malformations of the great toes. (e) Radiography of the right scapula and (f) right elbow shows abnormal calcification dependent on ligament ossification.

A Mexican family with one member affected by FOP was studied. The patient was a 19-year-old female. Her mother stated that the patient exhibited malformation of the great toes at birth. At the age of 8, the patient developed spontaneous and painful swelling in the right scapular area accompanied by a functional limitation of movement; her clinical laboratory abnormalities included increased serum alkaline phosphatase activity. Radiography demonstrated abnormal calcification and “pseudoexostosis” that was dependent on ligament ossification at the site of attachment to the long bones (Figure 1).

## 3. Mutation Analysis

ACVR1 617G>A mutation analysis was carried out with genomic DNA obtained from peripheral blood lymphocytes using DNAzol (GIBCO-BRL). Total genomic DNA was used as a template for PCR amplification (Select Cycler, Bio Products) with the following exon flanking primers: exon 4 forward 5′-CCA GTC CTT CTT CCT TCT TCC-3′ and reverse 5′-AGC AGA TTT TCC AAG TTC CAT C-3′; exon 6 forward 5′-GAC ATT TAC TGT GTA GGT CGC-3′ and reverse 5′-AGA GAT GCA ACT CAC CTA ACC-3′ and previously reported PCR conditions [2]. After amplification, the PCR products were digested with *Cac8I* and *HphI* restriction enzymes (New England Biolabs) for 1 h at 37°C and then submitted to 3% agarose gel electrophoresis. The undigested PCR products were prepared for sequencing using ultraclean PCR columns, in which a silica membrane assembly binds the DNA and allows the removal of primers, nucleotides, and enzymes. The purified PCR products of exon 4 were sequenced using the aforementioned primers in a genetic analyser (Applied Biosystems 3130 xl) that uses capillary electrophoresis and the ChromasPro v. 1.5 software for analysis. The sequences from the FOP patient were compared with those of her parents and unrelated healthy controls.

## 4. Ethics Considerations 

The clinical investigation was conducted according to the principles of the Declaration of Helsinki and was approved by the ethics committee of our institution. Written informed consent for genetic testing was obtained from the patient, her relatives, and controls.

## 5. Results

**Figure 2:**
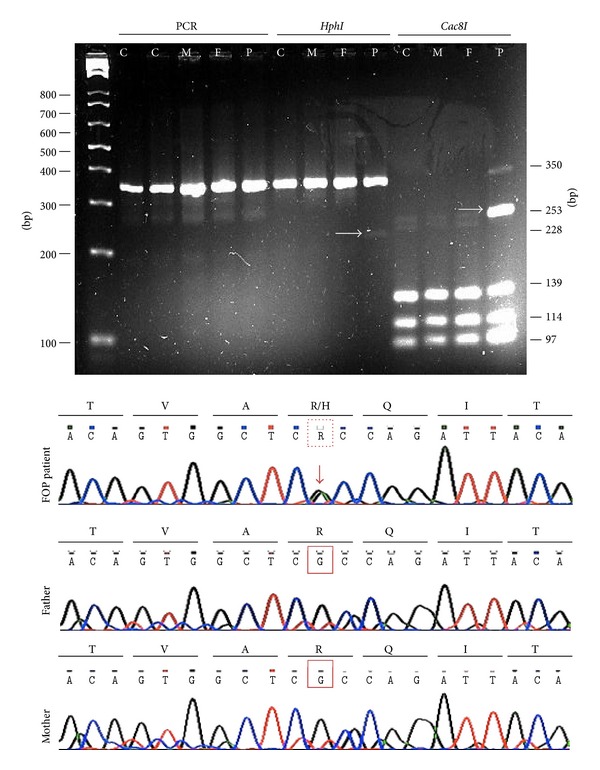
Genotyping in top panel: PCR amplification products were resolved by 3% agarose gel electrophoresis (C: control, M: mother, F: father, and P: FOP patient). As shown on the left, similar 350 bp products were amplified in controls, relatives, and the FOP case; however, after *Cac8I* digestion, a 253 bp fragment was observed exclusively in the FOP patient (marked with arrow). Bottom panel: a chromatogram showing the typical mutation at position 617, which causes an amino acid substitution of histidine for arginine at position 206 in the GS domain of ACVR1/ALK2.

PCR amplicons with primers specific to exon 4 produced similar 350 bp products in controls, relatives, and the FOP patient; however, *Cac8I* restriction digestion analysis produced a 253 bp fragment in the FOP patient only, and digestion with *HphI* produced an exclusive FOP fragment of 228 bp. The 253 bp *Cac8I* fragment was positive for the R206H (617G>A) mutation in exon 4 of the ACVR1 gene. The PCR analysis of exon 6 was negative for any mutation. Alignment of this sequence confirmed the ACVR1 617 mutation in our patient. This abnormality alters residue 206 of the protein such that an arginine is substituted by histidine. This change results in the dysregulation of BMP signalling. This case was classified as a *de novo *mutation because the ACVR1 206 mutation was not present in either of the patient's parents (Figure 2).

## 6. Discussion

A Mexican family with one member affected by spontaneous FOP was studied. The patient was a 19-year-old female when this molecular study was conducted; however, her first symptoms appeared at the age of 8. The patient exhibited clinical manifestations of repeated painful soft tissue inflammation and flare-ups, followed by progressive functional limitation in joint mobility. The patient was clinically diagnosed with FOP at the age of 10 and was treated with corticoids. The disease progression was not modified by this therapy. The patient lives in an isolated farming community and was referred to us by an orthopaedic surgeon for an opinion on whether to excise the bone exostoses. After clinical evaluation, the patient was informed about the difficulty of performing any surgery related to new bone formation and received counselling from the Center for Research in FOP and Related Disorders at the University of Pennsylvania School of Medicine in Philadelphia, PA, USA, through the guidelines of the international FOP consortium. This case was further studied with routine clinical laboratory and radiographic analyses, and the underlying molecular abnormality was defined and subsequently confirmed by genomic analysis in our laboratory, disclosing a mutation in her ACVR1/ALK2 gene. 

Fibrodysplasia ossificans progressiva is clinically characterised by malformations of the great toes and progressive heterotopic ossification [4]. The great toe malformations are a unique marker of this disease at birth; other symptoms appear within the first decade of life. In the reported case, as in the majority of FOP patients, the patient developed recurrent painful swelling of the soft tissue and inflammatory flare-ups, followed by the ossification of ligaments, tendons, skeletal muscles, and other soft tissues. FOP patients frequently develop a breastplate-like armour in the chest; this phenomenon caused some recurrent pneumonia in our patient [5, 6]. 

FOP is extremely rare, occurring in only one of every 2 million people [7]. In Mexico, little is known about this disease, and this particular clinical case gave us the opportunity to establish a molecular protocol for studying patients with this singular disease. 

Seminal studies of FOP by Kaplan et al. allowed a better understanding of FOP. These investigators characterised this enigmatic disease at the clinical, genetic, and molecular levels. They discovered that the ACVR1 617G>A mutation is responsible for the abnormalities observed in FOP. In addition, their contributions provided a better description of the roles of ACVR and BMP in bone formation. More recently, other mutations in the GS domain and kinase domain of ACVR1 have been described; these mutations are associated with atypical manifestations of FOP [8].

During FOP flare-ups, inflammation is mediated by macrophages, lymphocytes, and mast cells, which release granules that induce oedema in the involved muscles and ligaments. These events are followed by fibrogenesis and angiogenesis, which ultimately lead to ossification [9]. The pathophysiology of the inflammation in FOP seems to be related to the BMP binding that induces heteromeric complex formation by the type I and II BMP receptors. This clustering is followed by the transduction of downstream signals, including the *Smad* proteins, which in turn upregulate the transcription of target genes involved in inflammation, such as TNF, IL-1, IL-6, iNOS, chemokines, and other molecules [10–12]. 

Although there is no specific treatment for FOP, therapies aiming at reducing inflammatory flare-ups may help to improve patient quality of life by maintaining as much residual joint and muscle function as possible [13]. The use of corticosteroids for short periods during flare-ups improves symptoms, although animal models have shown that corticosteroids relieve inflammation but do not inhibit bone formation [14]. The use of bisphosphonates has been suggested as a potential therapeutic approach. Other new molecules may be able to block basic fibroblast growth factor and selectively block the BMP signalling pathway but are not yet available for clinical use [13].

In conclusion, we report a typical case of FOP with *de novo *ACVR1 mutation. To the best of our knowledge, this is the first FOP case to be genetically characterised in Mexico.
